# A Novel Murine Anti-Lactoferrin Monoclonal Antibody Activates Human Polymorphonuclear Leukocytes through Membrane-Bound Lactoferrin and TLR4

**DOI:** 10.1155/2015/285237

**Published:** 2015-11-15

**Authors:** Xiao-Min Hu, Yan-Rui Xu, Ru Yan, Shu-Liang Sun, Hong-Liang Dong, Jun Wang, Xiao-Ming Gao

**Affiliations:** Institutes of Biology and Medical Sciences, Soochow University, Suzhou, Jiangsu 215123, China

## Abstract

Soluble lactoferrin (LTF) is a versatile molecule that not only regulates the iron homeostasis, but also harbors direct microbicidal and immunomodulating abilities in mammalian body fluids. In contrast, little is known about the function of membrane-bound LTF (mbLTF), although its expression on human polymorphonuclear leukocytes (huPMNs) has been reported for decades. Given that LTF/anti-LTF antibodies represent a potential diagnostic/prognostic biomarker and a therapeutic target in patients with immune disorders, we wished, in the present study, to generate a novel human LTF- (huLTF-) specific mAb suitable for detailed analyses on the expression and function of mbLTF as well as for deciphering the underlying mechanisms. By using the traditional hybridoma cell fusion technology, we obtained a murine IgG1 (kappa) mAb, M-860, against huLTF. M-860 recognizes a conformational epitope of huLTF as it binds to natural, but not denatured, huLTF in ELISA. Moreover, M-860 detects mbLTF by FACS and captures endogenous huLTF in total cell lysates of huPMNs. Functionally, M-860 induces the activation of huPMNs partially through TLR4 but independently of phagocytosis. M-860 is thus a powerful tool to analyze the expression and function of human mbLTF, which will further our understanding of the roles of LTF in health and disease.

## 1. Introduction

Lactoferrin (LTF) is an 80 kDa glycoprotein of the transferrin family with iron-binding capabilities. In adult mammalians, LTF is mainly synthesized by glandular epithelial cells and secreted into the mucosal fluids that bathe the body surfaces [1, 2]. In addition to the evolutionally conserved iron-binding capacity, LTF plays diverse physiological roles as well. It regulates cellular growth/differentiation and represents a first-line host defense molecule against a broad range of microbial infections. Likewise, soluble LTF, through a variety of surface and/or intracellular receptors, modulates the functions of many immune cells and thereby influences the innate as well as the adaptive immunities [2–5]. Therefore, LTF has been considered as a diagnostic biomarker as well as a therapeutic target in many inflammatory and infectious diseases [5, 6].

Polymorphonuclear leukocytes (PMNs), or neutrophils, are the most abundant circulating leukocytes (constituting > 50%) in the bloodstream. Upon receiving appropriate signals, they leave the blood and migrate into the sites of infection/inflammation within hours, where they combat the invading microorganisms and eventually die. In the site of inflammation, activated PMNs are the main producers of LTF, which is normally stored in the secondary granules but released, together with other contents, during the degranulation process. Moreover, it has been reported that anti-LTF polyclonal Abs or purified IgGs from anti-neutrophil cytoplasm antibodies- (ANCA-) positive patients were capable of inducing the activation of human PMNs (huPMNs)* in vitro* [7–10]. Given the fact that huPMNs express membrane-bound LTF (mbLTF) as well, these data pointed out the presence of a previously unappreciated “reverse outside-in signal” mediated by mbLTF in modulating the function of huPMNs [11]. In this scenario, it is conceivable that this LTF-mediated activation of huPMNs will amplify local inflammation and thereby exacerbate tissue damage* in vivo* [12, 13]. As such, it is of importance to perform a systemic and detailed analysis on the expression and function of LTF on (immune) cells as well as to decipher the underlying mechanisms. To achieve this, a monoclonal antibody (mAb) targeting huLTF is far superior to polyclonal ones due to its high homogeneity and specificity. However, to our knowledge, no functional studies employing current commercially available anti-LTF mAbs have been reported [11].

In the present study, we, by using the traditional hybridoma technology, generated a novel mouse IgG1 (*κ*) mAb (herein referred to as M-860) against huLTF. Unlike other anti-LTF Abs targeting a linear epitope, M-860 appears to recognize a conformational epitope of huLTF while showing little cross-reactivity to bovine/murine LTF. In addition, it detects mbLTF on huPMNs by FACS analysis. More importantly, soluble M-860 is capable of triggering the activation of huPMNs, which is phagocytosis independent but partially abrogated by TLR4 inhibition. These data indicate that mbLTF, upon binding to its ligand, is capable of initiating signal transductions partially through TLR4 to modulate the function of huPMNs. Therefore, M-860 provides a powerful tool to analyze the expression and function of mbLTF on human cells.

## 2. Materials and Methods

### 2.1. Human Blood Samples

Human fresh blood was obtained from healthy adult donors with no sickness/medication during the past 10 days. Informed consent was provided in accordance with procedures approved by the local human ethics committee. Blood samples were immediately subjected to PMNs purification by Polymorphprep gradient centrifugation (Axis-Shield, Norseland) according to the manufacturer's protocol. PMNs were suspended in desired medium for downstream assays, and the purity/viability was typically >90% as evaluated by flow cytometry.

### 2.2. Mice and Immunization

Female BALB/c mice, 8–10 weeks of age, were purchased from the Model Animal Research Center (Nanjing, China). All animals were maintained under specific-pathogen-free (SPF) conditions and animal usage was conducted according to protocols approved by the Soochow University Institutional Animal Care and Use Committee.

To generate monoclonal Abs against human LTF (huLTF, purified from human milk, Sigma), female BALB/c mice were inoculated subcutaneously (s.c.) with 50 *μ*g huLTF which was dissolved in 50 *μ*L PBS and subsequently emulsified with an equal volume of complete Freund's adjuvant (CFA, Sigma, St. Louis, MO). Two weeks later, a booster injection was administered with 25 *μ*g huLTF emulsified in incomplete Freund's adjuvant (IFA, Sigma, St. Louis, MO). After 7 days, blood samples were taken by tail bleeding for the detection of huLTF-specific Abs. Animals with high anti-LTF Ab titers were boosted twice further with 25 *μ*g antigen in IFA at an interval of two weeks.

### 2.3. Cell Fusion and Hybridoma Selection

Three days after the last injection, splenocytes from immunized mice were isolated and fused with SP2/0 murine myeloma cells at a 5 : 1 ratio using PEG 1500, followed by culture in 96-well plate at 10^5^ cells/well with splenocytes from naive BALB/c mice as feeders (10^5^/well) in DMDM medium (Hyclone) supplemented with antibiotics and 10% FBS (Hyclone). After one week, supernatants were screened by ELISA and wells positive for anti-huLTF production were cloned and subsequently subcloned by limited dilution in HAT medium (Sigma). On day 14, hybridomas positive for anti-huLTF were further cultured by limited dilution in HT medium (Sigma), and from day 21 onwards, selected cells were cultured in DMEM medium. Eventually, a stable clone (hereafter referred to as M-860), exhibiting strong binding to huLTF in ELISA, was obtained.

### 2.4. LTF-Based ELISA

ELISA plate wells were coated with 2 *μ*g/mL recombinant human/bovine LTF (Sigma), murine LTF (lacking the first 27 amino acids, Sino Biological Inc.), or denatured huLTF (obtained by treatment with 1%  *β*-mercaptoethanol for 2 hrs or 95°C for 10 mins) in carbonate coating buffer (pH 9.6) overnight at 4°C. After five washes with PBST (PBS supplemented with 0.05% Tween 20), wells were blocked with 2% BSA (Amresco) in PBS for 2 hrs at 37°C before the incubation with serially diluted mouse sera or 1 *μ*g/mL purified Abs in PBS containing 0.1% BSA for 2 hrs at 37°C. After washing, wells were incubated with HRP-conjugated goat Abs against mouse/rabbit total IgGs (SouthernBiotech, Birmingham, USA), diluted 1 : 4000 in PBS with 0.1% BSA, at 37°C for 1 hr. Alternatively, goat Abs specific for mouse IgG1, IgG2a, IgG2b, or IgG3 (all from SouthernBiotech, Birmingham, USA) were used as indicated. Finally, a substrate solution containing o-phenylenediamine (OPD, Sigma) was added and signals were visualized at 492 nm by an ELISA plate reader.

### 2.5. Purification of M-860

BALB/c mice were injected intraperitoneally with pristine (0.5 mL/mouse) followed by the implantation of hybridoma cells (5 × 10^5^/100 *μ*L/mouse) 7–10 days later. After 7–12 days, ascites were collected, centrifuged, filtered, and subjected to purification of immunoglobulin G by protein G-sepharose affinity columns (GE Healthcare). The purity, specificity, and sensitivity of purified IgG were analyzed by SDS-PAGE electrophoresis or LTF-based ELISA, respectively.

### 2.6. Sequencing Analysis of the Variable Regions of the Heavy (VH) and Light (VL) Chains of M-860

Total RNA was isolated from 10^7^ hybridoma cells using RNAiso Plus and cDNA was synthesized with the oligo(dT) priming method (Takara). Primers used were as follows: VH, 5′-GGTBAARCTGVWGSAGTCTGG-3′ and 5′-TGGAGTTAGTTTGGGCAGCAG-3′; VL, 5′- ATGAGGTKCYYTGYTSAGYTYCTGRGG-3′ and 5′- TAACTGCTCACTGGATGGTGG-3′. PCR reactions were performed using pfu DNA polymerase (Takara). The amplification condition was 95°C for 5 min, followed by 35 cycles of 95°C for 30 s, 56°C for 1 min, and 72°C for 1 min, with a final extension of 10 min at 72°C. PCR products were electrophoresed through 1.5% agarose gels, and bands of interest were sliced out for DNA extraction followed by sequencing analysis.

### 2.7. Immunoprecipitation and Western Blot Analysis

Cells were washed with PBS and total proteins were extracted with RIPA buffer. Cell lysates were incubated with M-860, L3262 (a rabbit polyclonal Ab against LTF, Sigma), or corresponding controls (1 *μ*g) overnight at 4°C. Subsequently, protein G-sepharose was added followed by a further 1 hr incubation at 4°C. The precipitated proteins were subjected to SDS-PAGE in the presence of *β*-mercaptoethanol (2-ME). After transfer, the PVDF membrane was blotted with L3262 (5 *μ*g/mL) overnight at 4°C. After washing, the membrane was incubated with goat anti-rabbit IgG-HRP, followed by detection with ECL plus Western Blotting Detection system.

### 2.8. Conjugation of Antibodies with FITC

FITC-labeled Abs were prepared following the instructions provided by Sigma (St. Louis, MO). Briefly, 250 *μ*L of FITC (1 : 20 diluted in 0.1 M carbonate-bicarbonate buffer) was added dropwise to 1 mL antibody solution (5.0 mg/mL in 0.1 M carbonate-bicarbonate buffer, pH 9.0) while stirring the reaction vial, followed by incubation at room temperature for 2 hrs with gentle stirring. Unincorporated FITC molecules were removed through a 10 mL Sephadex G-25M column.

### 2.9. Culture of huPMNs

Human PMNs were cultured in RPMI-1640 (Hyclone) supplemented with 10% FBS (Hyclone) in the absence/presence of 10 *μ*g/mL LPS (Sigma), 10 ng/mL phorbol-12-myristate-13-acetate (PMA, Sigma), or different concentrations of antibodies at 37°C. After 1 hr, cells were collected and stained with a PE-coupled antibody recognizing an activated epitope of human CD11b (clone CBRM1/5, eBioscience) before FACS analysis. In some cases, PMNs were pretreated with phagocytosis inhibitor Dansylcadaverine (MDC, Sigma) for 30 mins or TLR4 inhibitor (CLI-095, InvivoGen)/CD14 neutralization Ab (clone 134620, R&D Systems) for 3 hrs before the addition of stimuli. After further incubation of 3 hrs, supernatants were harvested and stored at −20°C for the measurement of IL-8 by ELISA (Biolegend).

### 2.10. Measurement of Reactive Oxygen Species (ROS) by Flow Cytometry

ROS production by huPMNs was measured as described before [14]. Briefly, cells suspended in HBSS buffer with calcium, magnesium, and 5 mM D-glucose (5 × 10^5^/100 *μ*L in Eppendorf tubes) were incubated with 1 *μ*M dihydrorhodamine 123 (DHR123, Sigma) in the absence/presence of PMA (10 ng/mL) or M-860/L3262 as well as control Abs (50 *μ*g/mL) at 37°C for 1 hr. The reaction was stopped by placing the tubes in ice and cells were analyzed by conversion of fluorogenic substrate DHR123 to the fluorescent Rhodamine 123 (R123) in FACSCalibur (BD Biosciences).

### 2.11. Phagocytosis Assay

Human PMNs were incubated with LPS (10 *μ*g/mL) or M-860/control Abs (50 *μ*g/mL) at 37°C for 30 mins before the addition of FITC-labeled zymosan particles (Sigma). One hour later, samples were placed on ice for 5 mins followed by washing with ice-cold PBS. Percents of PMNs that had phagocytosed zymosan particles were analyzed by FACSCalibur. The analysis gate was restricted to PMNs based on their forward- and side-scatter properties to exclude free zymosan particles.

### 2.12. Confocal Microscopy

Human PMNs (5 × 10^6^/well) were incubated in chambers for live cell imaging precoated with 0.1% poly-L-lysine at 37°C for 60 mins to allow for cell adhesion. Cells were subsequently stained with optimally diluted antibodies against surface markers on ice for 60 mins, followed by fixation with 4% paraformaldehyde in PBS at room temperature for 20 mins before analysis by laser scanning confocal microscopy (Nikon).

### 2.13. Statistical Analysis

The Mann-Whitney paired test was used to compare the differences among groups by using GraphPad Prism 5.00 software (GraphPad, San Diego, CA), and values at *P* < 0.05 were considered significant.

## 3. Results

### 3.1. Generation of huLTF-Specific Hybridoma Clone M-860

To generate monoclonal Abs against huLTF, splenocytes from huLTF-immunized mice were fused with SP2/0 murine myeloma cells and cells were subsequently cultured in 96-well plates. Upon screening for the production of huLTF-targeting Abs by ELISA followed by cloning and subcloning by limited dilution, a stable hybridoma clone, termed M-860, was obtained.

### 3.2. Purified M-860 Is a Mouse IgG1 (*κ*) mAb That Recognizes huLTF with High Affinity

**Figure 1:**
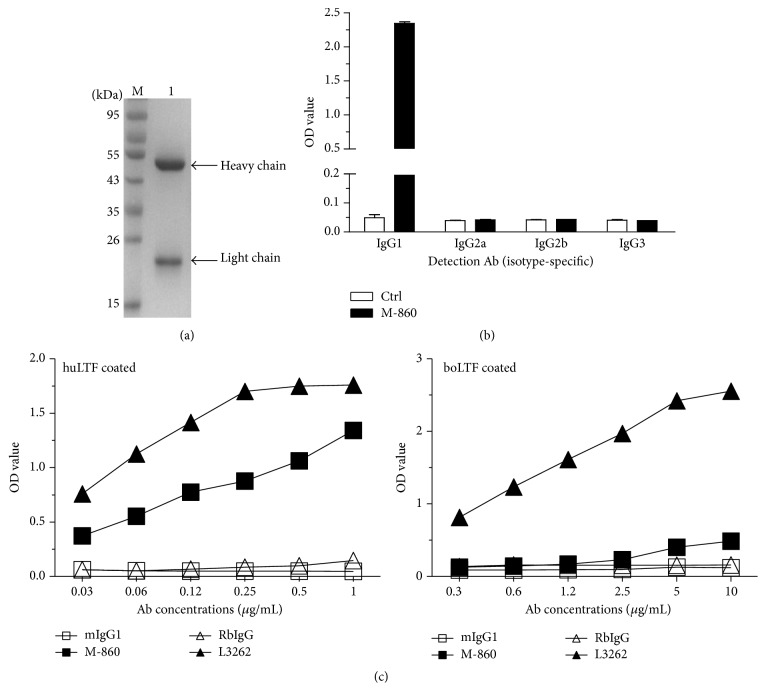
The purity, subtype, and specificity/affinity of M-860. Seven to twelve days after the implantation of hybridoma cells, ascites from BALB/c mice were collected and immunoglobulin G was purified by protein G-sepharose affinity columns. The purified IgG, M-860, was dialysed against PBS overnight at 4°C before the analysis for purity, subtype, and specificity/affinity by SDS-PAGE (a) or LTF-based ELISAs (b and c). (a) SDS-PAGE showing the purity of isolated M-860 antibody. Lane M: marker; Lane 1: purified M-860. (b) M-860 belongs to mouse IgG1 subtype and binds to huLTF in ELISA. M-860 (1 *μ*g/mL) or total mouse IgG (Ctrl) was incubated in ELISA wells precoated with huLTF for 2 hrs at 37°C. After washing, HRP-coupled goat Abs recognizing mouse IgG1, IgG2a, IgG2b, or IgG3 (indicated in *x*-axis) were added and incubated for 1 hr. Signals were visualized at OD 492 nm by an ELISA reader after the addition of OPD. (c) Line graphs showing the specificity and affinity of M-860 for huLTF. Different concentrations (as indicated in *x*-axis) of M-860, L3262, and their corresponding control Abs were incubated in ELISA wells precoated with huLTF (left panel) or bovine LTF (boLTF, right panel). Signals were visualized at OD 492 nm by an ELISA reader after sequential incubation with HRP-coupled goat anti-mouse/rabbit total IgG and then with OPD.

The obtained M-860 hybridoma cells were implanted into the peritoneum of mice for* in vivo* expansion and Ab enrichment. Seven to twelve days later, immunoglobulin G was purified from the ascites by protein G-agarose. As shown in Figure 1(a), the purified IgG exhibits high homogeneity in SDS-PAGE analysis as evidenced by the appearance of only two bands, corresponding to the heavy and light chains of mouse IgG, respectively (Figure 1(a)). Next, purified M-860 was incubated in ELISA wells precoated with huLTF, followed by detection with HRP-coupled goat Abs recognizing mouse IgG1, IgG2a, IgG2b, or IgG3 to identify its subtype. A vivid signal was only observed in wells containing both M-860 and goat anti-mouse IgG1 detection Abs (Figure 1(b)). We thus concluded that M-860 is a mouse IgG1 mAb capable of recognizing huLTF in ELISAs.

Given the high homology (~70%) among bovine, murine, and human LTFs, we wondered whether M-860 cross-reacts with bovine/murine LTF. To this end, titrated amounts of purified M-860 or mIgG1 were added to wells precoated with human, bovine, or murine LTF. After incubation and washing, the capture of Abs by precoated LTF was detected by HRP-conjugated goat anti-mouse IgG secondary Abs. In contrast to the dose-dependent binding of M-860 (0.03–1 *μ*g/mL) to huLTF-coated wells, no positive signals were detected in wells containing M-860 and bovine or murine LTF (Figure 1(c), data not shown). As expected, L3262, a rabbit polyclonal Ab against LTF, cross-recognizes human, bovine, and murine LTFs (Figure 1(c), data not shown). As the murine LTF used for coating here lacks the first 27 amino acids at the N-terminal, we could not exclude the possibility that M-860 targets an epitope located in this region of murine LTF. Nonetheless, our data indicate that M-860 is highly specific for huLTF with little cross-reactivity to bovine/murine LTF.

**Figure 2:**
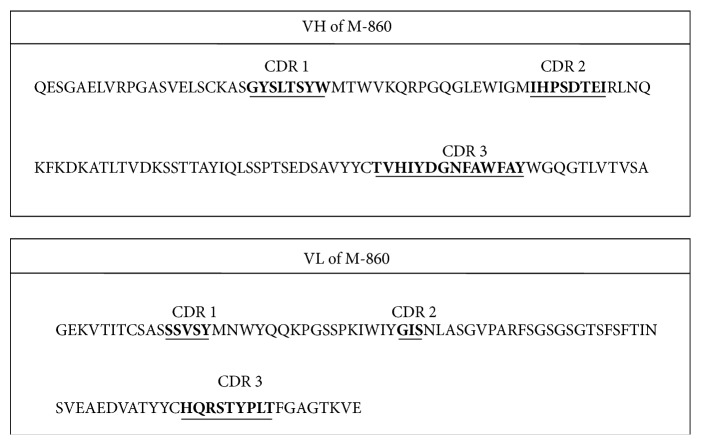
Sequencing analysis of the VH and VL of M-860. The variable regions of the heavy (VH) and light (VL) chains of M-860 were amplified by PCR as described in Section 2.6. After electrophoresis, bands of interest were sliced out for DNA extraction and sequencing analysis. Amino acid residues of the three complementarity-determining regions (CDR) were marked with underlines.

To further characterize M-860, its VH and VL were amplified by RT-PCR and sequenced, followed by alignment to the International MunoGeneTics Information System (IMGT; http://www.imgt.org/) data base.  Figure 2 shows the complementarity-determining region (CDR) sequence of VH and VL of M-860. Additionally, the alignment demonstrated that M-860 harbors *κ* light chain (data not shown). Based on these data, we concluded that M-860, a mouse IgG1 (*κ*) mAb, displays high specificity and avidity for huLTF.

### 3.3. M-860 Targets a Conformational Epitope of huLTF

**Figure 3:**
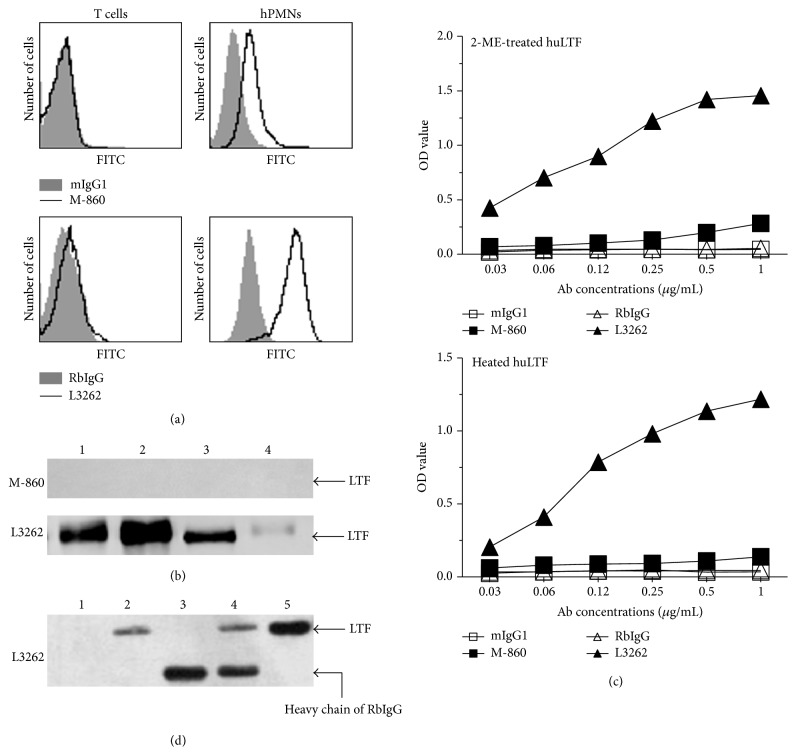
M-860 recognizes a conformational epitope of huLTF. (a) Representative histograms showing the staining of FITC-labeled M-860/L3262 on gated T lymphocytes or huPMNs. After lysis of erythrocytes, total leukocytes in human peripheral blood were stained with FITC-labeled M-860/L3262 before FACS analysis. FITC-labeled mIgG1/RbIgG were included as negative controls. Lymphocytes and huPMNs were gated according to their distinct forward- and side-scatter properties. (b) M-860 does not recognize huLTF in western blot analysis. Total cell lysates of huPMNs (2 *μ*g, Lane 1), recombinant human (50 ng, Lane 2), murine (200 ng, Lane 3), or bovine (200 ng, Lane 4) LTF were subjected to SDS-PAGE in the presence of 2-ME. After the transfer to a PVDF membrane, the membrane was blotted with M-860 or L3262 overnight at 4°C, followed by incubation with proper HRP-coupled secondary antibodies. (c) M-860 does not recognize denatured huLTF in ELISA. Serially diluted M-860/L3262 or corresponding control Abs (indicated in *x*-axis) were incubated in wells precoated with 2-ME-treated (1% 2-ME for 2 hrs, upper panel) or heat-treated (95°C for 10 mins, lower panel) huLTF, followed by routine ELISA procedures. (d) M-860 pulls down huLTF from total huPMNs lysates. Total cell lysates of huPMNs were incubated overnight with 1 *μ*g mIgG1 (Lane 1), M-860 (Lane 2), RbIgG (Lane 3), or L3262 (Lane 4) at 4°C, followed by incubation with protein G-sepharose. The precipitated proteins were subjected to western blot analysis with L3262 as the detection Ab. Recombinant huLTF (50 ng, Lane 5) was included as a positive control.

In addition to ELISA, flow cytometry and western blot analyses are two most commonly used techniques in protein studies; we thus tested the behaviors of M-860 in these assays. In line with previous reports showing the expression of mbLTF on huPMNs [11], huPMNs were positively stained with M-860 or L3262, while gated T lymphocytes were negative for both in parallel experiments (Figure 3(a)). Similar results were obtained on purified huPMNs with or without further* in vitro* stimulation by PMA (data not shown). These data indicate that M-860, like L3262, recognizes mbLTF on huPMNs. As such, M-860 is suitable for FACS analysis on the expression of mbLTF on cell surfaces.

Surprisingly, no positive signals were obtained with M-860 in western blot analysis using total huPMNs lysates, recombinant human, bovine, or murine LTF, while clear bands were visualized when the membrane was blotted with L3262 (Figure 3(b)). As the SDS-PAGE was conducted in reducing conditions (with 2-ME), we reasoned that denaturation of the protein may destruct the epitopes targeted by M-860 present in recombinant soluble huLTF (Figures 1(b) and 1(c)) as well as mbLTF on cell surface (Figure 3(a)). To test this, we first treated huLTF with heating or 2-ME before coating and then measured the ability of M-860 to recognize these denatured huLTF in ELISAs. In striking contrast to the dose-dependent binding of M-860 to natural huLTF (Figure 1(c), left panel), no binding of M-860 to wells coated with heat/2-ME-treated huLTF was observed (Figure 3(c)). As expected, the polyclonal Ab L3262 recognizes natural as well as denatured huLTF (Figures 1(c) and 3(c)). We thus concluded that treatment with heating or 2-ME destructs the epitope targeted by M-860 in huLTF.

To further confirm the capability of M-860 to capture huLTF, we incubated the total cell lysates of huPMNs with M-860, L3262, or control Abs overnight at 4°C in nonreducing buffers, followed by incubation with protein G-sepharose. The precipitated proteins were subsequently subjected to SDS-PAGE and detected by L3262. As shown in Figure 3(d), huLTF was visualized in proteins precipitated by M-860 or L3262, but not by control Abs (Figure 3(d)). We concluded that M-860 is capable of capturing endogenous huLTF in total cell lysates and thus can be used in immunoprecipitation assays.

So far, we have demonstrated that M-860 binds to natural (but not denatured) huLTF in ELISAs (Figures 1(c) and 3(c)), detects mbLTF on huPMNs (Figure 3(a)), and captures huLTF in total lysates in nonreducing conditions (Figures 3(b) and 3(d)). Together, our data indicate that M-860, a novel mouse mAb we generated, recognizes a conformational epitope of huLTF.

### 3.4. M-860 Induces the Activation of huPMNs

**Figure 4:**
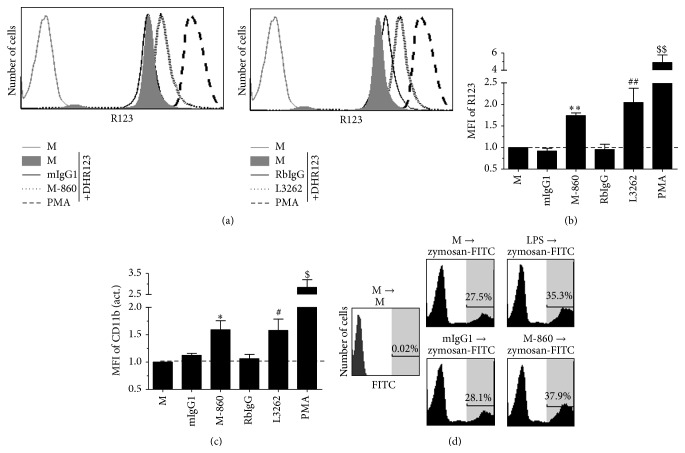
M-860 induces the activation of huPMNs. Purified huPMNs were incubated with PMA, M-860, L3262, or corresponding mIgG1/RbIgG control Abs in the presence (a and b)/absence (c) of DHR123 for 1 hr at 37°C before FACS analysis. (a) Representative histograms showing the increased production of ROS induced by LTF-specific Abs. ROS production was measured by the conversion of DHR123 to fluorescent R123. (b) Bar graph showing the mean fluorescence intensity (MFI) of R123 in cells. (c) M-860 upregulates CD11b expression (an activated epitope) on huPMNs. (d) M-860 enhances the phagocytosis activity of huPMNs. Cells were cultured in medium (M), LPS, M-860, or mIgG1 at 37°C for 30 mins before the addition of FITC-labeled zymosan particles. After 1 hr, cells were collected and the percent of PMNs phagocytosed zymosan particles was analyzed by FACS. Results were expressed as mean ± SEM of 9 (b) or 6 (c) different individuals. ^*∗*^ *P* < 0.05 versus M/mIgG; ^#^ *P* < 0.05 versus M/RbIgG; ^$^ *P* < 0.05 versus M; ^*∗∗*^ *P* < 0.01 versus M/mIgG; ^##^ *P* < 0.01 versus M/RbIgG; ^$$^ *P* < 0.01 versus M.

**Figure 5:**
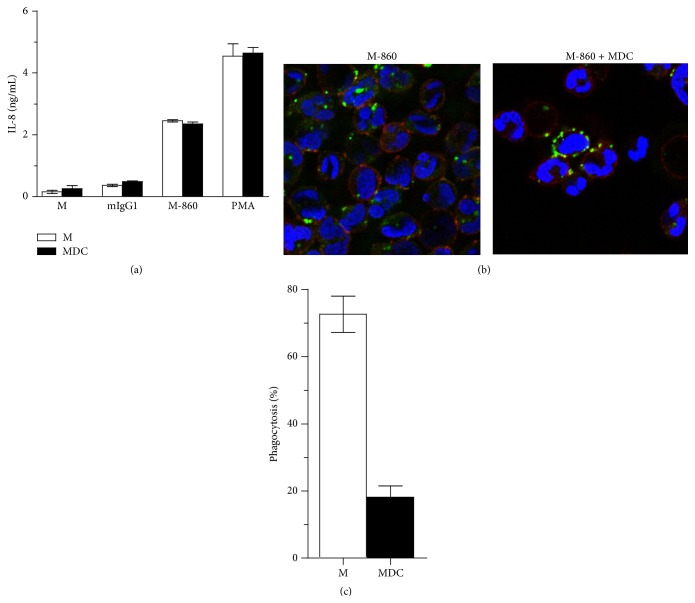
Phagocytosis-independent activation of huPMNs by M-860. (a) No effect of MDC on M-860-induced IL-8 production by PMNs. Cells were preincubated at 37°C with or without phagocytosis inhibitor MDC (25 *μ*M) for 30 mins before the addition of PMA, M-860, or mIgG1. Three hours later, levels of IL-8 were measured by ELISA. (b and c) MDC significantly inhibits the uptake of FITC-labeled M-860 by huPMNs. After the pretreatment with/without MDC (25 *μ*M) for 30 mins, FITC-coupled M-860 was added and cells were cultured for 1 hr further. Cells were fixed, stained with APC-coupled anti-CD11b (shown in red color, to visualized the cell membrane) before analysis by confocal microscopy. The percent of cells with intracellular FITC signal was quantified. Results were expressed as mean ± SEM of triplicates and represented at least two independent experiments.

Given that M-860 recognizes mbLTF on cells (Figure 3(a)), we next wished to test the functional relevance of this interaction. Human PMNs were isolated from peripheral blood by gradient centrifugation and then immediately cultured with soluble M-860, L3262, or corresponding isotype control Abs at 37°C before the measurement of surface CD11b levels as well as the production of ROS/IL-8 by FACS or ELISA. Mild but significantly increased production/expression of ROS/CD11b was observed in/on cells cultured with M-860 or L3262 (Figures 4(a)–4(c)). Moreover, coculture with M-860 resulted in greatly increased production of IL-8 by huPMNs (Figure 5(a)). Comparable percents of apoptosis were observed in cells incubated with medium, isotype controls, or M-860 for up to 24 hrs (data not shown).

We next determined whether M-860 could also augment the phagocytosis ability of huPMNs. Purified huPMNs were preincubated with M-860 or LPS for 30 mins at 37°C before the addition of FITC-labeled zymosan particles. After a further incubation of 1 hr at 37°C, the percent of PMNs positive for FITC signal was measured by FACS. As shown in Figure 4(d), pretreatment with M-860 or LPS, but not mIgG1, significantly increased the ability of PMN to take up zymosan particles. Moreover, M-860 slightly increased the formation of neutrophil extracellular traps (NETs) by huPMNs (data not shown). Based on the data presented in Figures 4 and 5(a), we concluded that soluble M-860 is capable of activating huPMNs.

### 3.5. M-860 Activates huPMNs in a Phagocytosis-Independent Manner

We next set out to elucidate the mechanisms underlying the activation of huPMNs by M-860. Given that huPMNs contains both membrane and granular LTF [11], we wished to determine whether M-860 induces the activation of huPMNs through binding to mbLTF and/or intracellular LTF. In the latter case, M-860 needs to be internalized by huPMNs first before binding to granular LTF to initiate signal transductions. Therefore, we first explored whether inhibiting the phagocytosis activity of huPMNs attenuates the production of IL-8 induced by M-860. Our data showed that pretreatment of huPMNs with different concentrations of a potent phagocytosis inhibitor, MDC, had no effect on IL-8 levels induced by M-860 or PMA (Figure 5(a), data not shown). The ineffectiveness of MDC in this assay could not be attributed to its inability in blocking phagocytosis as confocal microscopy analysis revealed that MDC greatly reduced the uptake of FITC-labeled M-860 by huPMNs (from 70% to 20%, Figures 5(b) and 5(c)). Together, these data indicate that mbLTF, but not intracellular LTF, mediates the activation of huPMNs induced by soluble M-860.

### 3.6.  M-860 Activates huPMNs Partially through TLR-4

**Figure 6:**
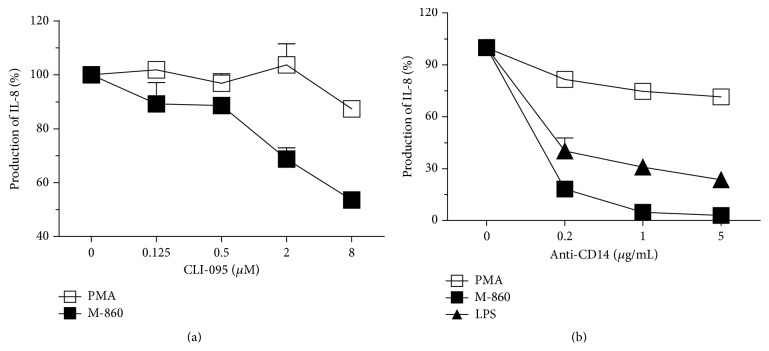
M-860 activates huPMNs through CD14 and TLR-4. Cells were preincubated at 37°C with different amounts of TLR-4 signaling blocker CLI-095 (a) or CD14 neutralization Abs (b) for 3 hrs before the addition of M-860. After further incubation for 3 hrs, levels of IL-8 were measured by ELISA. Levels of IL-8 in wells without CLI-095/anti-CD14 Ab were set as 100%. No inhibitions were observed with mIgG1 (isotype controls for anti-CD14, data not shown). PMA (10 ng/mL) and/or LPS (3 *μ*g/mL) were included as positive or negative controls. Results were expressed as mean ± SEM of triplicates and represented at least two independent experiments.

It has been reported that soluble LTF could bind to CD14 and signal via TLR-4 [15–17]. To test the possible involvement of TLR-4 in M-860 triggered huPMNs activation, we pretreated huPMNs with different concentrations of a specific TLR-4 signaling inhibitor, CLI-095, before the addition of M-860. As shown in Figure 6(a), addition of CLI-095 partially abrogated (~50% reduction at 8 *μ*M) the increased levels of IL-8 induced by M-860. Moreover, pretreatment of cells with an anti-CD14 neutralization Ab greatly reduced the binding of M-860 to cell surface (unpublished data) and thereby almost completely abolished M-860-induced productions of IL-8 (Figure 6(b)). It is unlikely that the stimulating effect of M-860 on huPMNs is mediated by contaminated LPS as the same batch of M-860 displays no stimulatory effect on murine macrophages and human THP-1 cells, both of which are very sensitive to LPS-mediated activation (unpublished data). In conclusion, our data indicate that mbLTF is associated with CD14 on the cell surface and consequently, upon occupation by its ligands like M-860, initiates signal transductions at least partially via TLR-4.

## 4. Discussion

In sharp contrast to the large body of reports on soluble LTF [2–4, 18–22], little has been advanced about the expression and function of mbLTF since its expression on huPMNs was reported decades ago [11, 23]. Here, by using M-860, an in-house generated novel mouse IgG1 (*κ*) mAb targeting a conformational epitope of huLTF, we demonstrated that mbLTF is capable of transducing a reverse outside-in signal partially through surface TLR-4 to modulate the function of huPMNs. Considering the increased levels of anti-LTF Abs in some patients with inflammatory disorders [12, 13], our study pointed out the presence of a previously unappreciated vicious circle mediated by mbLTF-anti-LTF interactions in exacerbating/perpetuating inflammatory reactions and tissue destructions in these patients. These data thus not only indicate that the expression and function of mbLTF on other human (immune) cells are worth further investigations, but also provide a powerful tool, mAb M-860 generated, for such studies.

In the hematopoietic system, LTF is mainly produced by PMNs. It is normally stored in the secondary granules of PMNs and is released upon degranulation. Accordingly, the mobilization of intracellular LTF to the cell surface of activated, but not resting, huPMNs was first observed by immunohistochemistry [23]. However, Deriy et al. detected mbLTF on both resting and activated huPMNs, but not monocytes, by FACS analysis using two different mAbs against huLTF [11]. By using the novel mAb M-860 generated in-house, we confirmed the expression of mbLTF on huPMNs with or without* in vitro* stimulation (Figure 3(a), data not shown). Given that huLTF does not possess transmembrane and cytoplasmic domains and is secreted from the cell after removal of the signal peptide [24, 25], it is conceivable that mbLTF is anchored to the cell surface via other membrane proteins, such as nucleolin and CD14 [26, 27]. Indeed, only a single band, identical to the molecular weight of recombinant huLTF, was detected in whole cell lysates of huPMNs by anti-LTF Abs in western blot analysis (Figure 3(d)). Furthermore, preincubation of cells with an anti-CD14 neutralization Ab greatly reduced, possibly by steric hindrance and/or inducing conformational changes of mbLTF, the binding of M-860 to cell surface, indicating that CD14 participates in the anchoring of mbLTF to the cell surface (unpublished data).

Soluble LTF is a multifunctional protein and participates in diverse pathophysiological processes. In addition to its well-known roles in iron homeostasis, LTF or its derivatives in body fluids exert a direct antimicrobial activity by binding to invading pathogens and/or cleaving arginine-rich bacterial sequences through a serine protease catalytic domain in its N-terminal [2, 20, 28]. Interestingly, LTF may blunt the anti-infectious immune responses by functioning as a decoy receptor for pathogen-associated molecular patterns (PAMPs), such as LPS and unmethylated CpG bacterial DNA, to diminish their immunostimulatory activities [2, 20]. Moreover, soluble LTF modulates the survival, growth, and function of many cells through a variety of surface and/or intracellular receptors [2–5, 13, 16, 17, 19–22, 29]. Our study revealed that huLTF exerts biological functions beyond its soluble form as mbLTF itself could serve as a surface receptor to modulate the function of huPMNs (Figures 4 and 5). In line with previous reports showing that soluble LTF binds to the CD14 molecule and could signal via TLR-4 [15–17], our data demonstrate that mbLTF is capable of transducing signals through complexing with CD14 and TLR-4, at least partially, to modulate the cellular function upon occupation by its ligands like M-860 (Figure 6). Given that soluble LTF binds to a panel of PAMPs derived from microorganisms, it is conceivable that the mbLTF interacts with PAMPs as well, and this interaction would facilitate the clearance of invading pathogens via augmenting the activation and function of huPMNs. Likewise, the occupation of mbLTF by anti-LTF Abs in ANCA-positive patients enhances the activation of huPMNs and thereby amplifies and perpetuates local inflammatory responses [12, 13, 30, 31]. Therefore, our data not only expand our knowledge on LTF, but also indicate that the expression and function of mbLTF merit further investigations, especially in patients with infectious or inflammatory diseases.

In conclusion, we have generated a novel mAb, M-860, against huLTF. Unlike current commercial anti-LTF Abs recognizing both natural and denatured huLTF, M-860 only binds to natural huLTF, indicating that it targets a conformational epitope. Moreover, upon occupation by soluble M-860, mbLTF is capable of triggering the activation of huPMNs through, at least partially, TLR-4. Using M-860 as a tool Ab, we are currently investigating the expression and function of mbLTF on other (immune) cells in humans.

## Abbreviations

LTF: Lactoferrin
huLTF: Human LTF
mbLTF: Membrane-bound LTF
PMNs: Polymorphonuclear leukocytes
huPMNs: Human PMNs
ANCA: Anti-neutrophil cytoplasm antibodies
VH: Variable regions of the heavy chains
VL: Variable regions of the light chains
2-ME: *β*-Mercaptoethanol
PMA: Phorbol-12-myristate-13-acetate
MDC: Dansylcadaverine
ROS: Reactive oxygen species
DHR123: Dihydrorhodamine 123
R123: Rhodamine 123
PAMPs: Pathogen-associated molecular patterns.
